# Experimental Zika virus infection of Jamaican fruit bats (*Artibeus jamaicensis*) and possible entry of virus into brain via activated microglial cells

**DOI:** 10.1371/journal.pntd.0007071

**Published:** 2019-02-04

**Authors:** Ashley Malmlov, Collin Bantle, Tawfik Aboellail, Kaitlyn Wagner, Corey L. Campbell, Miles Eckley, Nunya Chotiwan, Rebekah C. Gullberg, Rushika Perera, Ronald Tjalkens, Tony Schountz

**Affiliations:** 1 Arthropod-Borne and Infectious Diseases Laboratory, Department of Microbiology, Immunology and Pathology, College of Veterinary Medicine and Biomedical Sciences, Colorado State University, Fort Collins, Colorado, United States of America; 2 Department of Environmental and Radiological Health Sciences, College of Veterinary Medicine and Biomedical Sciences, Colorado State University, Fort Collins, United States of America; 3 Veterinary Diagnostic Laboratories, Department of Microbiology, Immunology and Pathology, College of Veterinary Medicine and Biomedical Sciences, Colorado State University, Fort Collins, Colorado, United States of America; US Department of Agriculture, UNITED STATES

## Abstract

The emergence of Zika virus (ZIKV) in the New World has led to more than 200,000 human infections. Perinatal infection can cause severe neurological complications, including fetal and neonatal microcephaly, and in adults there is an association with Guillain-Barré syndrome (GBS). ZIKV is transmitted to humans by *Aedes* sp. mosquitoes, yet little is known about its enzootic cycle in which transmission is thought to occur between arboreal *Aedes* sp. mosquitos and non-human primates. In the 1950s and ‘60s, several bat species were shown to be naturally and experimentally susceptible to ZIKV with acute viremia and seroconversion, and some developed neurological disease with viral antigen detected in the brain. Because of ZIKV emergence in the Americas, we sought to determine susceptibility of Jamaican fruit bats (*Artibeus jamaicensis*), one of the most common bats in the New World. Bats were inoculated with ZIKV PRVABC59 but did not show signs of disease. Bats held to 28 days post-inoculation (PI) had detectable antibody by ELISA and viral RNA was detected by qRT-PCR in the brain, saliva and urine in some of the bats. Immunoreactivity using polyclonal anti-ZIKV antibody was detected in testes, brain, lung and salivary glands plus scrotal skin. Tropism for mononuclear cells, including macrophages/microglia and fibroblasts, was seen in the aforementioned organs in addition to testicular Leydig cells. The virus likely localized to the brain via infection of Iba1^+^ macrophage/microglial cells. Jamaican fruit bats, therefore, may be a useful animal model for the study of ZIKV infection. This work also raises the possibility that bats may have a role in Zika virus ecology in endemic regions, and that ZIKV may pose a wildlife disease threat to bat populations.

## Author summary

The rapid spread of Zika virus through a naïve population in the Americas resulted in novel and severe disease manifestations, including fetal and neonatal microcephaly, and GBS. These disease complications make understanding the pathology and ecology of ZIKV a priority. Captive Jamaican fruit bats were challenged with ZIKV to determine their susceptibility, to assess whether bats may play a role in virus ecology, and if they might serve as an animal model to better understand ZIKV pathophysiology. The bats became acutely infected and mounted an antibody response. Three terminally euthanized inoculated bats had antibody titers of 3200, 28 days PI. Evidence of virus replication and associated pathologies were found in the brain, testes, lungs and salivary glands of some of the inoculated bats. The virus showed predilection for mononuclear cells, including resident Iba1^+^ macrophage/microglial cells, and Leydig cells.

With no discernible disruption to the blood brain barrier nor distribution of viral antigen indicative of circumeventricular neuroinvasion, microglia cells may be a possible route of entry of ZIKV into brains of bats. Further investigations are needed to determine the mechanisms of neuroinvasion of ZIKV in bats, further determine feasibility of bats as an alternative animal-model for congenital Zika syndrome, and what role bats might play in ZIKV viral ecology.

## Introduction

Zika virus (ZIKV) was first isolated from a sentinel rhesus macaque in Uganda in 1947 and subsequently from *Aedes africanus* mosquitoes in the same location [1]. The first human cases were identified in 1954 in Nigeria and serosurveys found evidence of a broad geographic distribution for ZIKV throughout Africa and Asia with sporadic cases in humans [2, 3]. The first recognized ZIKV epidemic occurred in Yap State, Federated State of Micronesia in 2007. An estimated 73% of residents were infected, and of those 18% presented with clinical disease [4]. In 2013, a second epidemic occurred in French Polynesia with 28,000 cases reported. During the latter outbreak, the incidence rate of Guillain-Barré syndrome (GBS) increased 20-fold and first indication of a connection between ZIKV infection and GBS was established [5]. The virus spread to Brazil in 2015 [6, 7] and has since disseminated throughout much of tropical South America, Central America, the Caribbean, and the southern United States, with more than 200,000 confirmed cases [8]. ZIKV can also cause congenital Zika syndrome (CZS) in naïve populations and is therefore a virus of high concern [3].

Zika virus is maintained in an urban cycle, transmitted between an *Aedes* mosquito vector and humans thereby maintaining endemicity [9]. It is generally accepted that the virus transmits between non-human primates and vectors in a sylvatic cycle; however, the sylvatic cycle has not been well characterized in the Old World and little is known about a New World sylvatic cycle [9, 10]. Molecular analysis of ZIKV to better understand viral phylogenetics suggests that animal hosts affected viral evolution and therefore may play an important role in viral ecology [11].

In the 1950s and ‘60s, the susceptibility of bats to ZIKV was investigated. Shepherd and Williams [12] screened 172 wild bats from 12 different species in Uganda for antibodies against ZIKV and found 16/44 little free-tail bats (*Tadarida pumila*) and 26/36 Angolan free-tail bats (*T*. *condylura*) were seropositive by hemagglutination inhibition assay. Additionally, two Angolan free-tail bats were experimentally inoculated with ZIKV and serially bled to test for viremia. Both animals were viremic on days 2, 4 and 6 as determined by paralysis in mice inoculated with the sera from those two bats [12]. Simpson and O’Sullivan [13] experimentally inoculated three straw-colored fruit bats (*Eidolon helvum*), three Egyptian fruit bats (*Rousettus aegyptiacusi*), and five Angolan free-tail bats. Two of the straw-colored fruit bats were viremic and had seroconverted. One of the Egyptian fruit bats was viremic and two had seroconverted. The Angolan free-tail bats were euthanized on days 1, 3, 5, 7 and 10 days post inoculation and screened for viral tropism. At one day post infection, a kidney was trace positive [13]. Finally, Reagan et al. [14] inoculated 20 New World little brown bats (*Myotis lucifigus*) by 5 different routes: intracranial, intraperitoneal, intradermal, intrarectal and intranasal. Bats in all groups, with the exception of the intranasal group, developed fatal neurological disease 4–7 days post inoculation. Brain tissue was virus-positive in all animals with clinical disease, determined by inoculation of mice with brain homogenate suspension [14].

Considering the evidence that African bats are naturally susceptible to ZIKV and that little brown bats develop disease, the question emerged: could bats serve as a natural reservoir host for ZIKV in the New World? To test this hypothesis, we inoculated Jamaican fruit bats (*Artibeus jamaicensis*), among the most abundant bats in the Caribbean, Central America and Mexico, with ZIKV to examine virology, immunology and pathology of the infection. Although virus was detected in several organs, including the testes and brains, no overt clinical signs were detected, and substantial viremia or viruria was not evident. These results suggest that Jamaican fruit bats are unlikely to serve as amplification hosts but that ZIKV infection may constitute a wildlife disease threat to bats.

## Results

### Experimental infections

Bats for this project were obtained from the Colorado State University breeding colony approved by the Institutional Animal Care and Use Committee (protocol 16-6512A). Two experimental infections were conducted; a pilot study and a time course study. In the pilot-study, three male bats (AJ-z7, AJ-z8, AJ-z9) were intradermally inoculated with 7.5x10^5^ plaque forming units (pfu) ZIKV, strain PRVABC59; a high dose to assess susceptibility. No signs of disease were apparent during this 28 day experiment; however, all three bats had antibody titers of 3200 on day 28 (Table 1). After demonstration of susceptibility in the pilot study, a time course study was conducted. Six male bats (AJ-z1 through AJ-z6) were identically inoculated and two were euthanized at 2, 5 and 10 days post inoculation (dpi). No conspicuous signs of disease were observed in any of the inoculated bats. Necropsies immediately followed euthanasia and no significant gross pathology was evident.

**Table 1:** Individual titers in 28 dpi bats inoculated with ZIKV by ELISA.

Animal ID	Titer
AJ-z7	3200
AJ-z8	3200
AJ-z9	3200
Pos Control (convalescent human sample)	≥12800
Neg Control (uninfected bat)	Neg

### Detection of viral RNA in urine and brain

**Fig 1:**
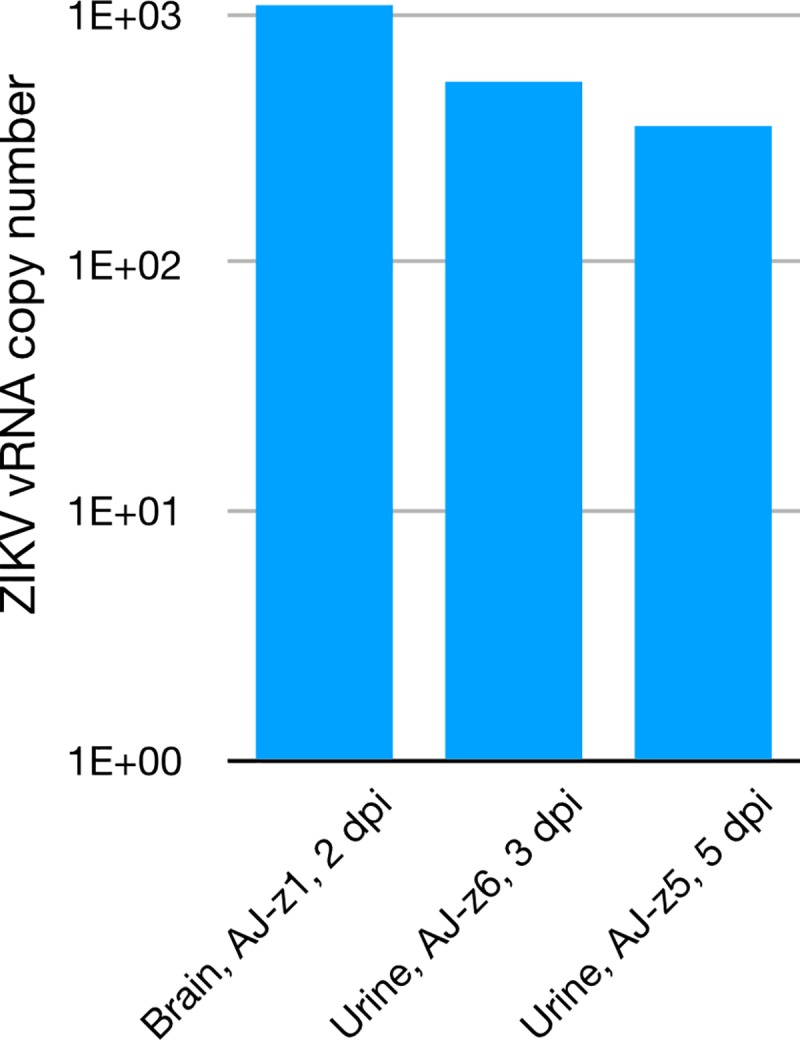
Zika virus RNA abundance in infected Jamaican fruit bats. Viral RNA was detected in the brain one bat (AJ-z1) euthanized on day 2 and in the urine collected from two other bats (AJ-z6, AJ-z5) on days 3 and 5 post infection.

Quantitative probe-based reverse transcription PCR (qRT-PCR) was performed on serum-inoculated Vero cell supernatants, serum, brain, lung, liver, spleen, kidney, urinary bladder, prostate and testes from bats from both studies. In addition, urine collected during the time course study was similarly assayed. Urine from bats AJ-z6 at 3 dpi and AJ-z7 at 5 dpi had low levels of vRNA whereas bat AJ-z1, euthanized at 2 dpi, had low levels of vRNA in its brain (Fig 1). All other samples were negative. Sera from AJ-z2 at 2 dpi, and AJ-z3 and AJ-z4 at 5 dpi were negative by ELISA. Sera were blind passaged on Vero E6 cells in an attempt to isolate ZIKV and all were negative for cpe and PCR.

### Histopathology

#### Hematoxylin and Eosin Stain (H&E)

Heart, lung, liver, kidney, testes, prostate, urinary bladder, and brain were collected from all 9 animals as well as salivary glands from 3/9 bats. All samples were blindly read by one pathologist. A summary of the consistent histopathology findings is listed in Table 2.

**Table 2:** Histopathological findings in bats inoculated with ZIKV.

Days post inoculation		2 dpi		5 dpi		10 dpi		28 dpi (pilot study)		
Bat #		AJ-z1	AJ-z2	AJ-z3	AJ-z4	AJ-z5	AJ-z6	AJ-z7	AJ-z8	AJ-z9
**Lungs**	Pneumonia	+/-	+/-	+/-				+/-		+/-
	Infiltrates and microscopic hemorrhage				+/-	+/-				
**Heart**	Cellular infiltrates		+/-							
	Cardiomyocyte necrosis					+/-	+/-	+/-		+/-
**Testes**	Degeneration and lymphocyte infiltration				x				x	
**Brain**	Neuronal degeneration						x	x		x
**S. glands**	Salivary gland inflammation			x						

Lesions in the lungs, heart, and kidney may be incidental as similar lesions have been seen in other experiments with different infectious agents and in negative control animals, indicated by “+/-”. Lesions in the testes, brain and salivary gland (sal. gl.) likely induced byZIKV infection, indicated by an “x”.

For the time course study, AJ-z1 at 2 dpi showed mild pulmonary congestion with multifocal areas of interstitial pneumonia, mild intra-alveolar hemorrhage and mild atelectasis. Terminal airways had slightly increased amounts of mucus. Kidneys had multifocal interstitial infiltrates of small numbers of lymphocytes. All other tissues were within normal limits. In AJ-z2 at 2 dpi, lungs showed milder pathology than AJ-z1 with minimal interstitial to perivascular infiltrates predominately lymphocytes and macrophages with a band of collapsed air spaces subjacent to the pleural surface. There were focal lesions in the left ventricle of the heart where there was individual cell loss or else fragmentation of the sarcoplasm of scattered cardiomyocytes. Degenerate/necrotic cardiomyocytes were accompanied by infiltrations of small numbers of macrophages, lymphocytes and satellite cells. All other tissues were within normal limits.

Lungs from AJ-z3 at 5 dpi had minimal focal interstitial histiocytic pneumonia with atelectasis. Kidneys showed multifocal chronic lymphohistiocytic pyelitis with a few degenerate and detached epithelial cells accumulating in the renal pelvis and infiltration of pelvic stroma by small numbers of mixed inflammatory cells. Mandibular salivary gland showed focal moderate cellular infiltrates of periductular lymphocytes and macrophages. Affected salivary ducts contained detached and degenerate epithelial cells and leukocytes. Occasional ducts were encircled by granulation tissue and a few heterophils. Rare apoptosis was evident in the lining epithelium of such ducts. All other tissues were within normal limits. AJ-z4 at 5 dpi had lungs with minimal alveolar septal infiltrates scattered within collapsed lung parenchyma along with multifocal microscopic hemorrhages. Kidneys had multifocal areas of mineralization. In the outer medulla and at the cortico-medullary junction were rare perivascular infiltrates of lymphoplasmacytes. Esophagus and lymphoid tissue associated with palatine salivary gland showed focal mild lymphoplasmacytic inflammation. Moderate numbers of lymphocytes and plasma cells were arranged in columns parallel to the respiratory mucosal epithelium of the nasophayrnx. The lumen contained increased amounts of mucus and a few inflammatory cells, mainly heterophils and lymphocytes. In the testicles, there was focal testicular degeneration manifested by presence of giant spermatids in the lumina of affected seminiferous tubules and accumulation of a small numbers of interstitial lymphocytes and macrophages. All other tissues were within normal limits.

**Fig 2:**
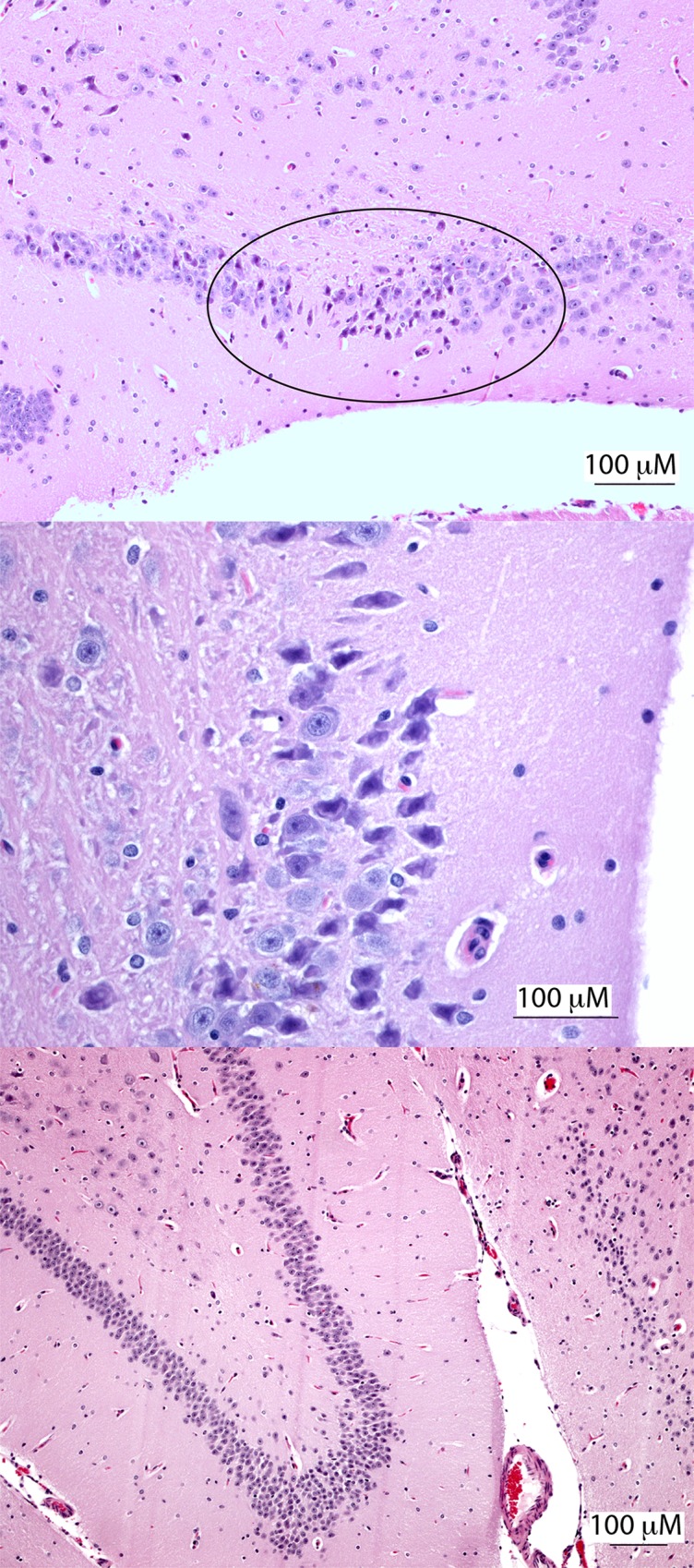
Hippocampus from AJ-z6, 10 dpi compared to negative control Jamaican fruit bat (H&E). (A) AJ-z6, 10 dpi area CA3 of the hippocampus with focal pyrimidal neuronal necrosis, circled. (B) AJ-z6, 10 dpi 400x magnification of lesion from figure A demonstrating angular, pyknotic nuclei, and hypereosinophilic cytoplasm of necrotic neuronal cell bodies. (C) Negative control bat hippocampus showing even and homogenous pyrimidal neuronal populations in all layers of hippocampus.

Lungs from AJ-z5 at 10 dpi had minimal interstitial to perivascular infiltrates with multifocal atelectasis and microscopic hemorrhages. The left papillary muscle of the heart showed rare multifocal cardiomyocyte necrosis characterized by rounding up of individual cardiomyocytes. Necrotic cardiomyocytes appeared with hypereosinophilic cytoplasm, devoid of cross striations or fragmented and rarely vacuolated. Minimal interstitial hypercellularity due to increased activity of satellite cells and infiltration of small numbers of lymphocytes was observed in the vicinity of degenerate/necrotic cardiac muscle fibers. Kidneys had an area of focal lymphoplasmactyic pyelitis. Additionally, there was a focal area of mineralization and inflammation in the inner medulla. All other tissues were within normal limits. AJ-z6 at 10 dpi had occasional focal inflammation and cardiomyocyte degeneration in the left ventricle and interventricular septum. Area CA3 of the hippocampus in the brain showed focal pyrimidal neuronal necrosis with a focal area of mineralization around a vessel in the cerebral cortex along with focal gliosis and individual neuronal necrosis (Fig 2). All other tissues were within normal limits. Testicular, neural and salivary glands’ lesions are believed to be associated with ZIKV infection as they were not seen with other viral infections.

**Fig 3:**
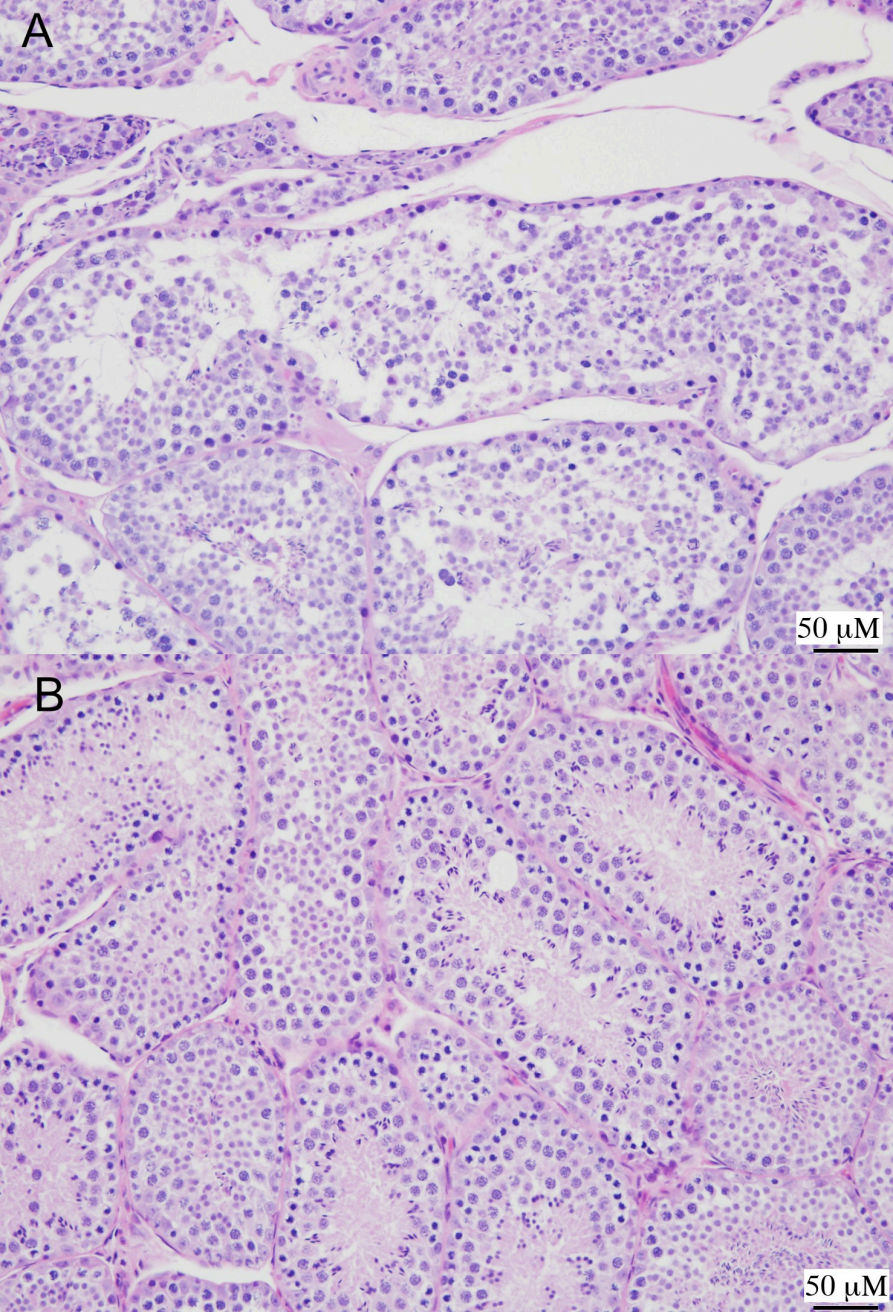
Testes from AJ-z8, 28 dpi compared to negative control Jamaican fruit bat (H&E). (A) Focal testicular degeneration. Seminefrous tubules are disorganized with no mature sperm in lumen and and multifocal accumulation of luminal giant spermatids. (B) Negative control bat normal testes.

In the pilot study bats, AJ-z7 at 28 dpi had more prominent interstitial pneumonia with congestion of the lungs compared to earlier time points. The heart had minimal cardiomyocyte degeneration and necrosis with hypercellular interstitium and increased amounts of mature fibrous connective tissue. The kidney had focal interstitial infiltrates of the cortical and outer medullary interstitium. The brain showed degenerate neurons in area A3 of the hippocampus. All other tissues were within normal limits. AJ-z8 had minimal focal testicular degeneration (Fig 3). All other tissues were normal. AJ-z9 had perivascular lymphocyte pulmonary infiltrates and atelectasis. Heart demonstrated locally extensive lymphocytic and histiocytic pericarditis. Kidneys showed multifocal interstitial lymphocytic infiltrates. Brain had focal, perivascular infiltrates of small numbers of lymphocytes at the subfornical commissure. The reticular formation showed multifocal neuronal degeneration/necrosis.

#### Immunohistochemistry and immunofluorescence

**Fig 4:**
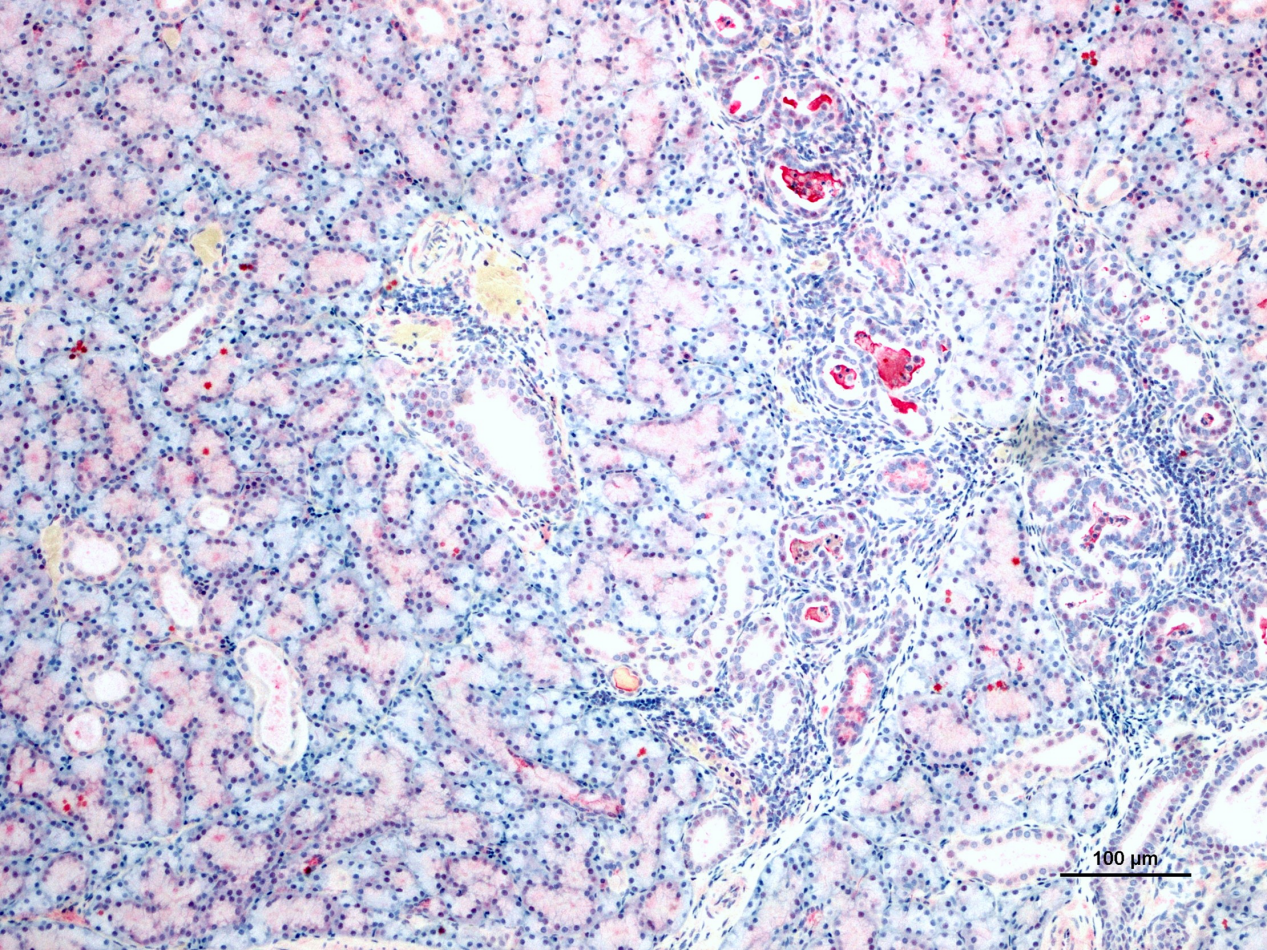
Salivary gland from AJ-z3, 5 dpi, IHC photomicrograph for ZIKV antigen in salivary gland of infected bats. Luminal immunoreactivity of the salivary gland showing multifocal periducular inflammation and accumulation of viral antigen in degenerate ductular epithelium and/or leukocytes.

**Fig 5:**
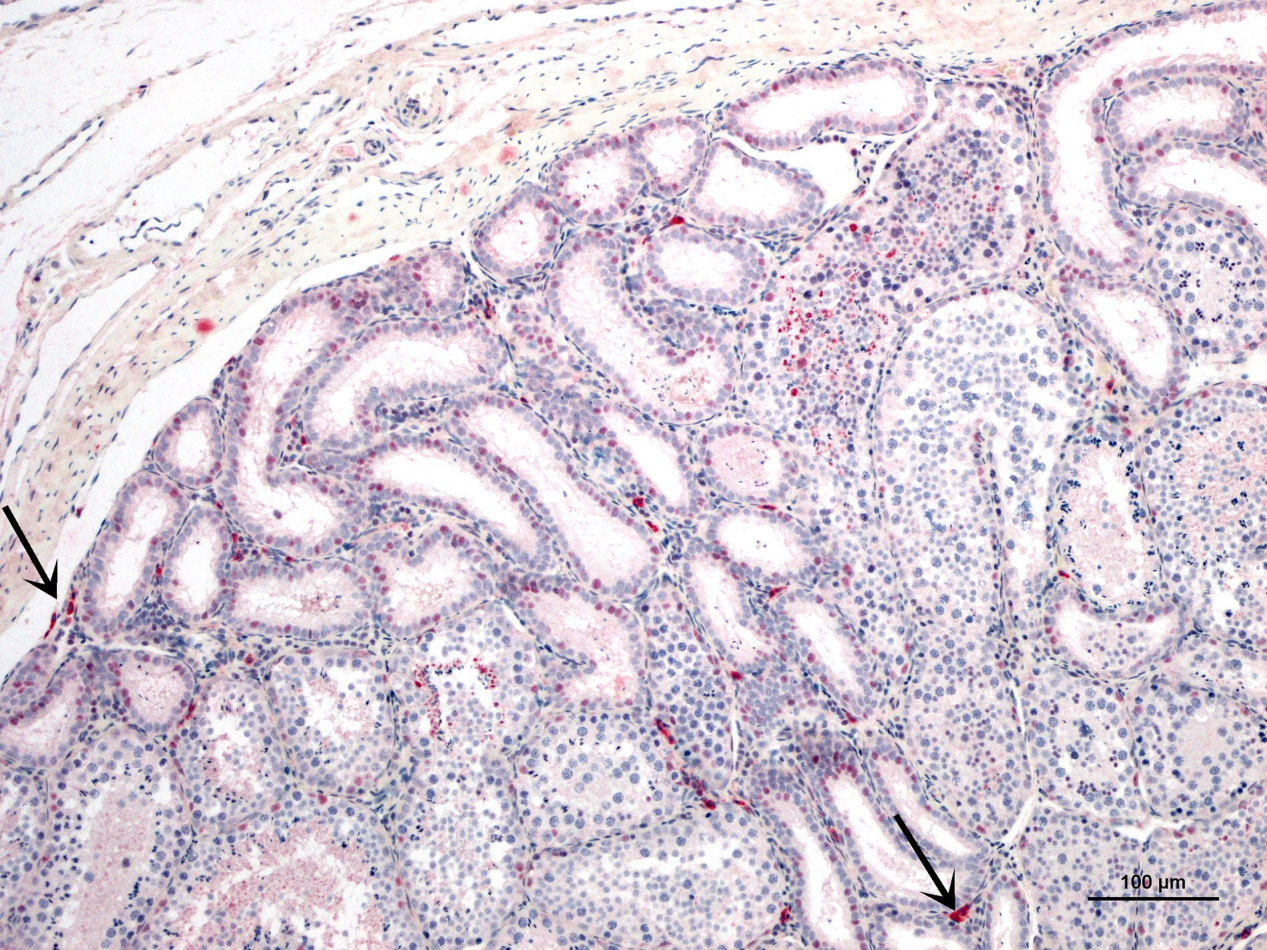
Testes from AJ-z5, 10 dpi, IHC photomicrograph for ZIKV antigen in testicles of infected bats. Mononuclear cell immunoreactivity, arrows highlighting mononuclear cells consistent with macrophages and Leydig cells.

**Fig 6:**
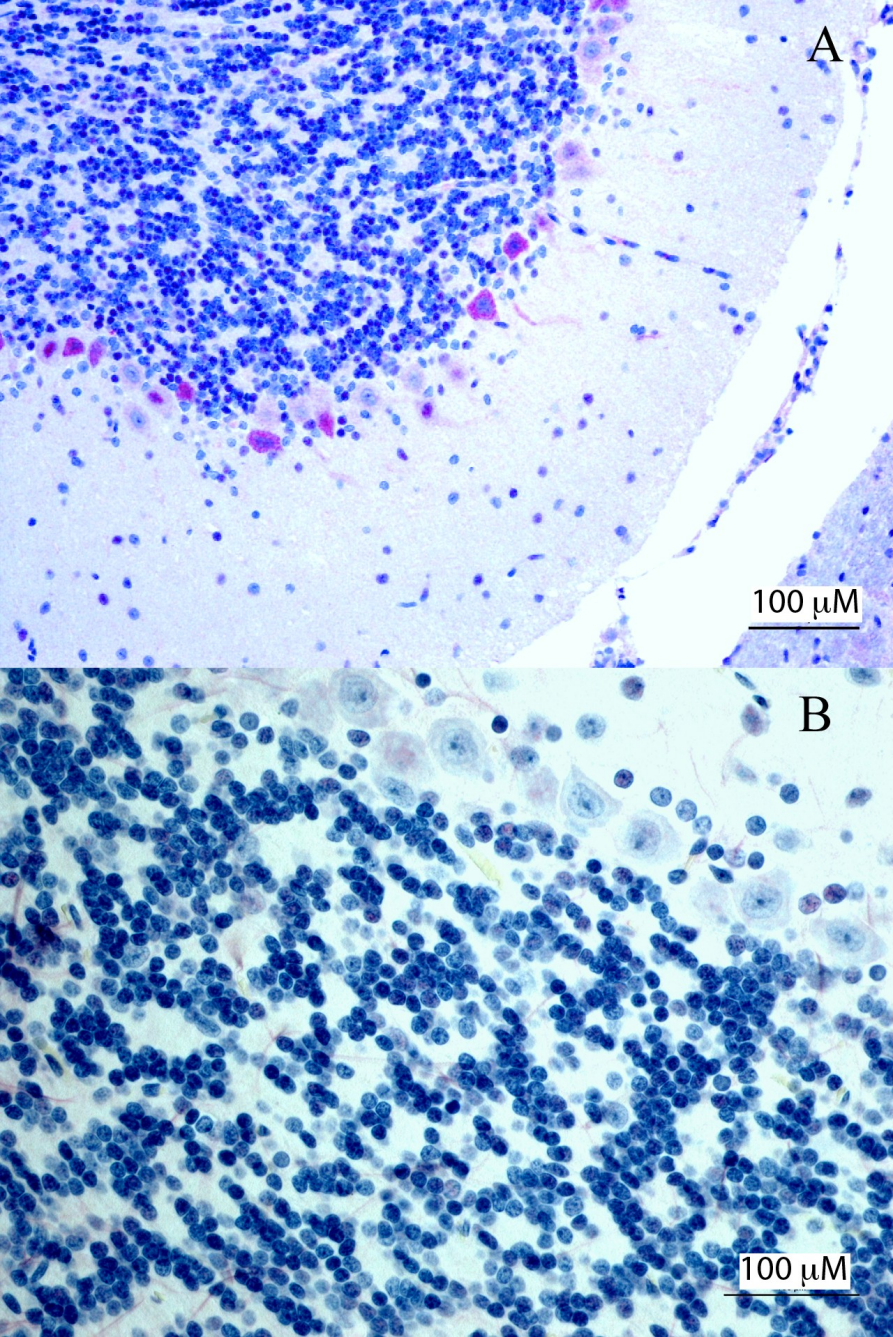
Immunoreactivity in AJ-z5, 10 dpi cerebellum, polyclonal anti- ZIKV antibody highlighting viral antigen in cerebellum of infected bats compared to negative control Jamaican fruit bat. (A) Multifocal Purkinje cell immunoreactivity in AJ-z5. (B) Negative control cerebrellum showing no immunoreactivity in Purkinje cells.

**Fig 7:**
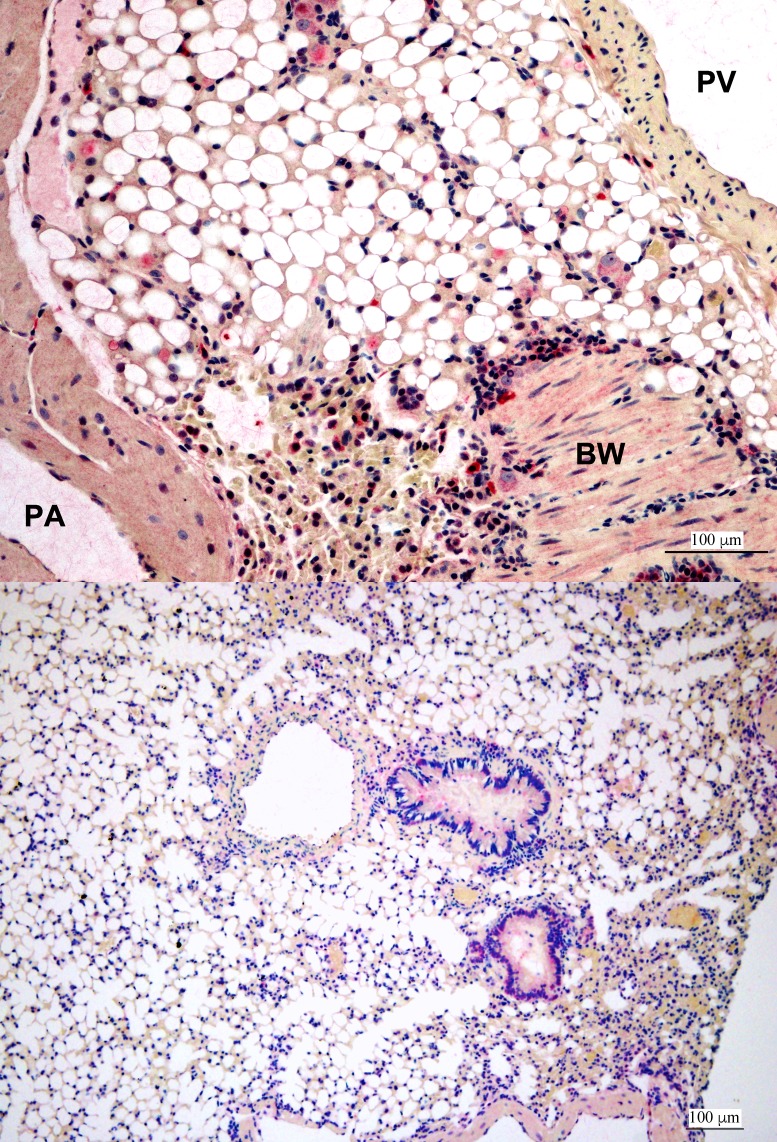
Immunoreactivity in AJ-z8, 28 dpi lung, IHC photomicrograph for ZIKV antigen in lungs of infected bats compared to negative control Jamaican fruit bat. (A) Hilum of the lung shows immunoreactivity in mononuclear cells consistent with macrophages and fibroblasts around the pulmonary artery. PA, pulmonary artery. PV, pulmonary vein. BW, bronchiolar wall. (B) Negative control without immunoreactivity.

**Fig 8:**
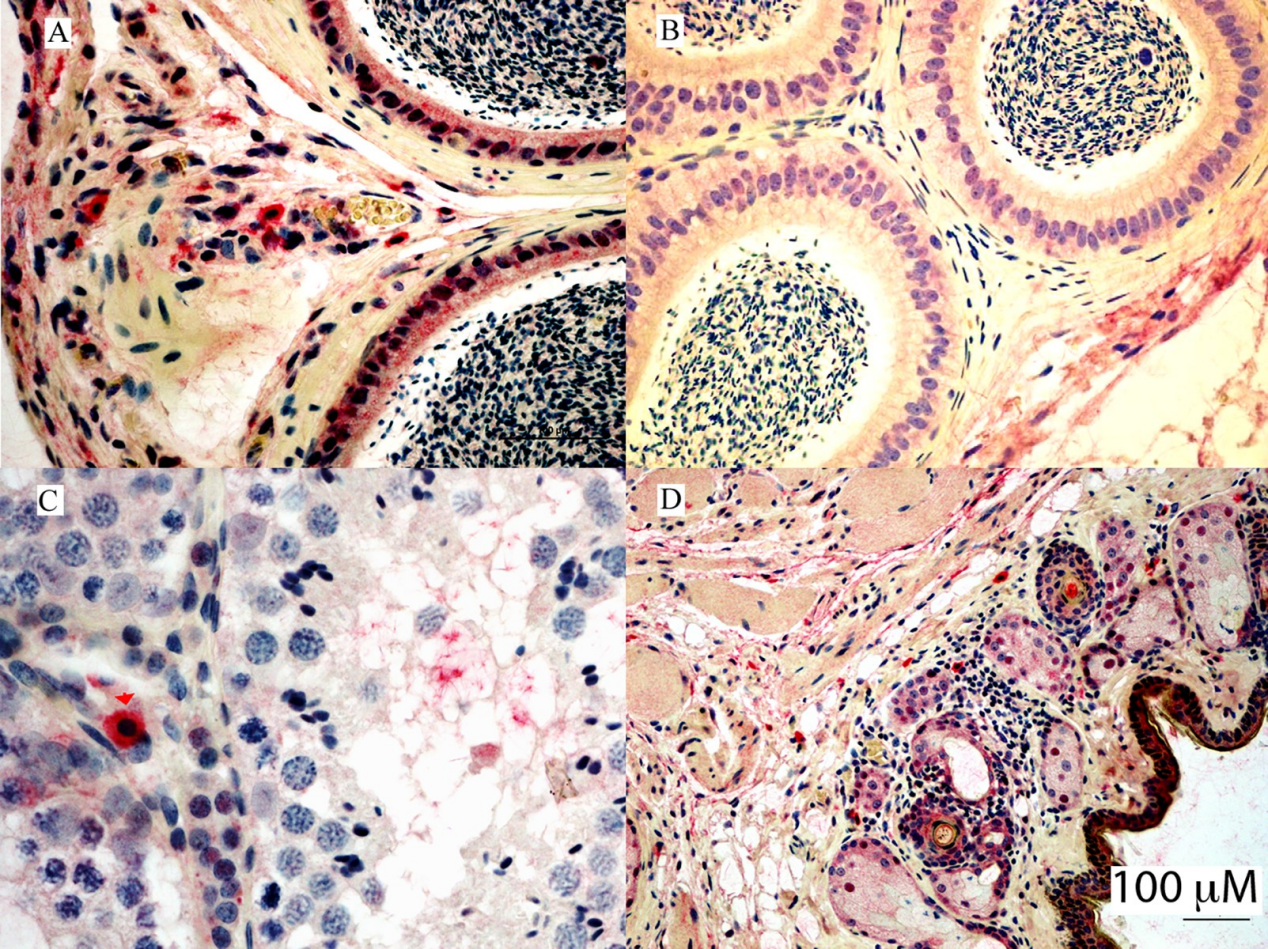
Immunoreactivity in AJ-z8, 28 dpi testes, IHC photomicrograph for ZIKV antigen in testicle and scrotum of infected bats compared to negative control Jamaican fruit bat. (A) Tunica albuginea perivascular immunoreactivity mostly in macrophages and fibroblasts of AJ-z8. (B) Negative control without immunoreactivity. (C) Interstitial immunoreactive mononuclear cells consistent with macrophages (black long arrows) and Leydig cells (short red arrows) in AJ-z8. (D) Focal lymphocytic dermatitis and immunoreactive mononuclear cells in AJ-z8.

Tissues were stained with a polyclonal antibody for ZIKV (CDC, Fort Collins). AJz-3 at 5 dpi with inflammation of the mandibular salivary gland had moderate immunoreactivity in the lumen of affected ducts (Fig 4). AJ-z5 at 10 dpi had immunoreactive cells in the brain and mononuclear cell immunoreactivity in the testes (Fig 5). Additionally, AJ-z5 demonstrated immunoreactivity in purkinje cells of the cerebellum (Fig 6A). AJ-z8 at 28 dpi had immunoreactive cells around the pulmonary arteries in the lungs (Fig 7A). AJ-z8 also had immunoreactivity perivascullarly in the tunica albuginea of the testes (Fig 8A). Scrotal skin had focal lymphocytic dermatitis with immunoreactive mononuclear cells (Fig 8D). Cell morphology consistently identified mononuclear cells compatible with macrophages and fibroblasts as the primary cell types showing immunoreactivity against ZIKV antigen.

**Fig 9:**
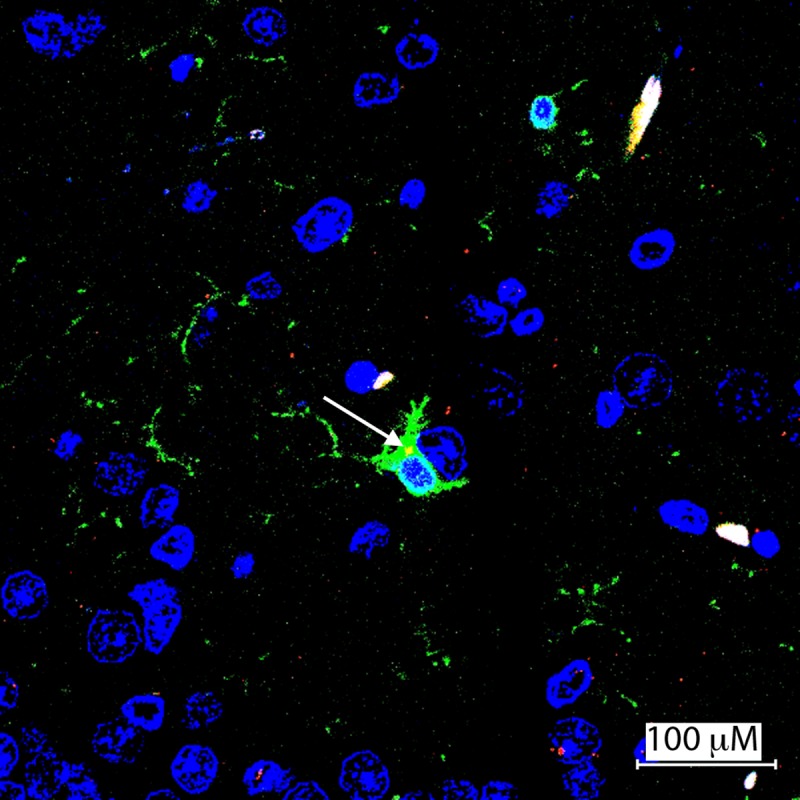
Confocal photomicrograph of ionized calcium binding adaptor molecule (Iba 1)—labeled microglial cells in cerebrum of AJ-z5, 10 dpi. A confocal Z-stack merged image depicting Iba-labeled microglial cell (green) with its characteristic processes and intracytoplasmic ZIKAV antigen co-localization (yellow).

Brain and testicular tissues stained with both goat polyclonal goat anti-Iba1 (green) and monoclonal 4G-2 flavivirus E specific antibodies (red) showed co-localization (yellow) of ZIKV antigen in cytoplasm of activated microglial cells with their characteristic morphology in the cerebral cortex of infected bats 10 dpi in the time course study and 28 day dpi in the pilot study (Fig 9). Increased microgliosis was noted in the vicinity of co-localization sites. The gliosis was also prominent in the cerebellum and hippocampus especially around dead neurons. In the testicles, occasional macrophages showed similar co-localization similar to that noted in the brain in the testicular interstitium, inner layer of tunica albuginea and scrotum. Cells consistent in morphology with Leydig cells were similarly highlighted by ZIKA viral antigen only showing strong immunoreactivity using polyclonal anti-ZIKV antibody.

## Discussion

Two bat infection experiments were conducted in this investigation; 1) a pilot study to determine susceptibility of Jamaican fruit bats to ZIKV infection, and 2) a time course study to better understand pathophysiology and chronology of events pertaining to the dynamics of viremia, viral tropism, replication and shedding of the virus in a New World bat species. The goal was to determine whether bats can be used as an animal model for ZIKV pathogenesis and to assess the possible role of bats in ZIKV ecology in the New World.

In the pilot experiment, no signs of disease were apparent during the 28-day study. Sera collected at euthanasia indicated modest antibody titers of 3200 for each bat by ELISA (Table 1), whereas the human -convalescent control serum titer was ≥12,800. Bats typically have low to modest antibody titers, perhaps due to limited somatic hypermutation and affinity maturation [15–22].

Concerning viremia, cell-serum supernatants, blind passage supernatants, and neat serum results were all negative. Although serum is routinely used for ZIKV diagnostics in humans, it may not be the most suitable sample [23–27]. In one investigation ZIKV patient had negative serum sample for the duration of the study, whereas whole blood yielded positive qRT-PCR results from days 9 to 101 [27]. One possible explanation for the phenomenon of negative serum in human patients is that the virus during acute infection disseminates via a cell-associated viremia or as novel findings suggest that the virus gets phagocytized in neutrophils and therefore whole blood is a more sensitive diagnostic sample than serum.

Viruria is commonly detected in ZIKV-infected humans [26]; therefore, urine may be an equally important diagnostic sample with higher viral load in early infection when compared to blood in humans and other primates [23–26]. Although urine collection from bats was challenging, we collected urine from some of the inoculated bats in the time course study. AJ-z6 exhibited viruria only at 3 dpi, and AJ-z7 was equivocal only at 5 dpi, corroborating the findings in other mammals that urine may be a route of viral shedding early in infection. Urine from one human patient was positive from the first time point (6 dpi) through 14 dpi and again on day 56. Similarly, saliva from that same patient was positive from day nine through day 14 and again on day 49 [27]. Another investigation compared diagnostic samples of 80 infected patients and showed that urine was positive in 50 of them, whereas serum was only positive in 19 patients by qRT-PCR. The study concluded that viral loads in urine were ten-fold higher compared to serum and that uremia lasted longer [25]. These data corroborated the first study that identified ZIKV shed in urine in which there was a higher viral load in urine for longer duration compared to serum [26]. ZIKV RNA in plasma was detected in the bats by qRT-PCR between 2 and 6 dpi, but between 2 to 17 dpi in urine [26]. The lack of detectable viremia in the serum of bats is congruent with some of the human and NHP investigations in that viremia is low and short-lived. Detached renal pelvic urothelial cells and degenerate salivary gland ductular epithelium as seen in the current study will make urine and saliva equally important fluids to collect in order to maximize detection of ZIKV in the acute and established stages of infections.

For this experiment, all male bats were used because female bats are prioritized for colony expansion. ZIKV exhibited tropism for the testes with strong immunoreactivity in reproductive organs (Figs 4 & 7). Histologically, minimal focal testicular degeneration in two bats (Fig 2) suggests viral related pathology may be minimal. In humans it has yet to be completely elucidated what reproductive organs harbor ZIKV, it has been determined that semen contains ZIKV both in both vasectomized and unvasectomized men [27, 28]. This suggests that ZIKV is sequestered in the testes and/or accessory sex glands. Mouse models have demonstrated ZIKV infection and associated pathology in the testes [29–31] of humanized BLT mouse model with infection primarily targeting macrophages and Leydig cells [32]. Limited investigation has been done relating to infection of accessory sex glands in mouse models, but one study that assessed the prostate found no virus, possibly due to differential expression of the receptor candidate in the testes but not in the prostate [29]. For this experiment the finding of viral antigen and viral RNA in the testes but not in the prostate is consistent with published animal models and may suggest the potential for bats to serve as another animal model.

Three bats had histopathological alterations in the hippocampus at later time points and one bat had viral nucleic acid present in the brain as determined by qRT-PCR demonstrating tropism for the CNS, a tissue predilection also documented in humans and animal models. ZIKV has a predilection for nervous tissue in animal studies and disease manifestation in humans. As a neurological teratogen, ZIKV has been detected in the brain mononuclear cells in human newborns with fatal microcephaly and fetal miscarriages. Histological lesions are varied but may include parenchymal calcification, microglial nodules, gliosis, cell degeneration, mononuclear infiltration and necrosis [33–35]. In non-human animal models, evidence for viral tropism has been found in brain and/or peripheral nervous tissue [36–39]. In immunocompromised mouse models, the virus has a predilection for the brain but with the mice engineered for specific immune traits it is difficult to know to what extent this recapitulates natural ZIKV pathophysiology [40]. In the bats used in this experiment, evidence of ZIKV-induced pathology in the brain is consistent with what has been seen in human newborns and fetuses.

The novel finding of co-localizing ZIKV antigen in bat Iba1^+^ microglial/macrophage cells lends support to the earlier evidence of microglial cell infection via Axl ligand bridging ZIKV particles to glial cells [41]. Iba1 (aka, allograft inflammatory factor 1, Aif1) is a microglia/macrophage-specific calcium-binding protein, which has actin-bundling activity that participates in membrane ruffling and phagocytic activity of activated microglia. Activated microglial cells appeared with increased ability of cell migration and phagocytosis, which is controlled by remodeling of membrane cytoskeleton [42]. The morphology of cells with co-localization in the brain of infected bats is consistent with activated microglia depicting prominent branched processes. Recent primate models in rhesus and cynomolgus macaques demonstrated similar viral distribution of ZIKV antigen to that in bats, described herein. High-level of ZIKV was evident in cerebellar neurons and the same studies documented involvement of Iba1 positive microglial cells in CNS infections. In primate models there is increasing evidence that ZIKV antigen was detected in individuals with the highest peak plasma viremia, which in part implies that ZIKV may initially seed the CNS by a passive spillover from circulating monocytes to resident microglial cells. This is further substantiated in all of human and animal studies, which did not show any evidence of disruption to BBB or viral distribution reminiscent of circumventricular distribution seen in alphavirus animal models [43].

In addition to brain and testes immunoreactivity, scrotal skin and mandibular salivary gland also harbored viral antigen. Distribution of viral antigen in bat tissues suggests that infection in this species recapitulates human infection, which is thought to start with infection of epidermal and dermal cells with subsequent dissemination to multiple organs including salivary glands as viral RNA can be detected in human saliva [44, 45]. The histopathology for AJ-z5, 5 dpi showed sialoadenitis and the presence ZIKV antigen by IHC (Fig 3). This suggests ZIKV may be shed in the saliva, although additional animal experiments need to be performed to confirm such a route of shedding. The results presented here suggest that Jamaican fruit bats may be a suitable animal model for examining ZIKV infection to elucidate its pathogenesis. Jamaican fruit bats may also serve as a model to ascertain sexual transmission, in utero transmission, teratogenesis and neurological pathophysiology. It may be that ZIKV is a wildlife disease threat for bats that could lead to infertility in some males, which could impact bat populations.

ZIKV is thought to be maintained in two different distinct cycles: sylvatic—cycling between non-human primates (NHP) and mosquito species, and urban—cycling between humans and mosquito species [3]. While there are limited data on what mosquito species feed on Jamaican fruit bats, evidence for natural flavivirus infection has been identified in wild New World bats. Dengue virus (DENV) RNA and antibodies to DENV were detected in multiple species of bats, including Jamaican fruit bats, in Mexico [46]. Additionally, antibodies to DENV were detected in multiple bat species including those of the *Artibeus* genus in Costa Rica and Ecuador [47, 48]. These data indirectly provide evidence for mosquito-bat interactions in the wild; either through consumption of bat-blood meals taken by mosquitoes or bat consumption of infected mosquitoes.

As it pertains to a wildlife reservoir, wild NHPs have antibody to ZIKV including several monkey species trapped near Ziika Forest [10], and wild and semi-captive orangutans in Borneo [49]. Not only have NHP been found to be seropositive, but also many other mammals, including rodents, horses, cows, and goats [50, 51]. Furthermore, experimental inoculation of various North American species resulted in seroconversion (cottontail rabbits, boar goats, pigs, and leopard frogs) and demonstrated viremia (nine-banded armadillo and leopard frogs) [52]. Molecular epidemiology suggests animals play an important role in an enzootic cycle [11]. Much about the enzootic cycle of ZIKV has yet to be understood but it stands to reason that bats may be capable of maintaining the virus in nature. Jamaican fruit bats are found in northern South America, Central America, and the Caribbean—areas that now have ZIKV potentially exposing bat populations to the virus [8, 53]. However, the data presented here suggest it is unlikely that Jamaican fruit bats can serve as amplification hosts of ZIKV, unless virus sequesters in some as-yet unidentified way that could lead to periodic shedding of virus. It may also be that some bats become persistently infected and can transmit sexually to maintain virus within populations of bats. Further experimental and field studies will be necessary to fully understand the ecological role of bats in ZIKV maintenance.

## Materials and methods

### Ethics statement

All animal procedures were approved by the Colorado State University (CSU) Institutional Animal Care and Use Committee (protocol 16-6512A) and were in compliance with U.S. Animal Welfare Act.

### Bats

CSU has a captive colony of Jamaican fruit bats (*Artibeus jamaicensis*), a neotropical fruit bat indigenous to much of South America, Central America and the Caribbean [53]. Colony bats are kept in a free flight room measuring 19’w x 10’l x 9’h. Roosting baskets are hung from the ceiling throughout the room and drapes of different cloth material are positioned for hanging and roosting. Ambient temperature is maintained between 20°C and 25°C, with humidity between 50% and 70%, and a 12 hour light/12 hour dark light cycle via a computer-controlled system. Diets consist of a combination of fruits (Shamrock Foods, Fort Collins, CO), Tekald primate diet (Envigo, Huntington, UK), molasses, nonfat dry milk and cherry gelatin that are placed in multiple feeding trays around the room once a day. Fresh water is provided. In addition, fruit is hung around the room to stimulate foraging behavior and serve as enrichment.

For infection experiments, bats were trapped using a butterfly net and placed in an 20”d x 12”w x 18”h cage for 24 hours prior to inoculations to allow for acclimation. Hanging clothes were provided for roosting and coverage. Food and water are placed in open trays in the bottom of the cage and changed daily. Tray liners were changed every two days, and cages and hanging clothes are changed every two weeks. Due to the social nature of these bats, minimums of two bats were kept in cages at all times to mitigate potential stress.

### Experimental inoculations

Two sets of experiments were performed; a pilot study and a time course study. Zika virus strain PRVABC59. PRVABC59 was isolated in 2015 by Centers for Disease Control and Prevention (Fort Collins, CO) from an infected individual who traveled to Puerto Rico (GenBank accession no. HQ234499). The virus stock titer is 3x10^7^ plaque forming units (pfu) per ml of media, and the fourth passage was used for both studies.

For the pilot study, three male bats were anesthetized with 1% to 3% isoflurane to effect with an oxygen flow rate of 1.5 L/min, administered with a gas mask. Animals were placed on a heating pad to maintain body temperature and respirations continuously monitored. The dorsum of each animal was disinfected with 70% ethanol and 25ul containing 7.5x10^5^ p.f.u of virus was administered subcutaneously (sc) at the level of the scapula with a sterile hypodermic 25 gauge needle in a biosafety cabinet. When procedures were finished, bats were removed from isoflurane and placed back in the cage in ventral recumbency. Respirations were monitored until animal was fully awake and ambulated normally. Bats were identified as AJ-z7, AJ-z8 and AJ-z9. Animals were euthanized at 28 days post-inoculation (dpi).

For the time course study, six male bats were anesthetized under the same protocol as the pilot study. Animals were placed in ventral recumbency. After disinfecting the dorsum of each animal with 70% ethanol, 0.15mls of 1% lidocaine was administered sc at the level of the last rib with a 25 gauge sterile hypodermic needle as a local anesthetic. IPTT300 transponders (BioMedic Data Systems, Inc., Seaford, DE) were inserted sc at the level of the caudal edge of the scapula. Twenty-five microliters containing 7.5x10^5^ p.f.u of virus was administered sc at the level of the cranial edge of the scapula. Recovery followed the same protocol as for the pilot study bats. Animals were identified as AJ-z1 through AJ-z6. AJ-z1 and AJ-z2 were euthanized at two dpi. AJ-z3 and AJ-z4 were euthanized at 5 dpi. AJ-z5 and AJ-z6 were euthanized at 10 dpi.

Female bats were excluded from the study because they are prioritized for breeding to sustain and expand upon the colony.

### Monitoring

For the pilot study, bats were visually monitored twice daily for fourteen days, and then monitored once a day for an additional fourteen days. For the time course study, bats were monitored twice a day throughout the experiment. For both studies, energy levels, behavior, ability to ambulate, respirations, presence of oral or nasal discharge, and fecal consistency were all assessed.

### Urine collection

During the time course study urine was collected at 2, 3, 5 and 10 dpi from as many bats as possible. Urine was collected by allowing bats to grasp screen cloth with their feet and then the bat was placed in a clear solo cup (Dart Container, Lake Forest, IL) with the screen covering the top of the cup as a lid, and kept in place with a rubber band. This allowed the bats to hang in a clear container. Bats were monitored for 45 minutes. If they urinated, bats were removed from the collection contraption and placed back in the cage without disrupting the urine. Urine collection was attempted on all remaining bats at each time point, but not all bats would urinate at each collection attempt. Urine was successfully collected as follows: two dpi from AJ-z3 and AJ-z4; three dpi from AJ-z3, AJ-z5 and AJ-z6; five dpi from AJ-z3, AJ-z4, AJ-z5 and AJ-z6; and ten dpi from AJ-z5 and AJ-z6. Urine was pipetted off the surface of the cup with a sterile pipette tip and put in a 1.5 ml microcentrifuge tube and stored at -80°C for future use. Urine volume ranged between 5 ul and 15 ul.

### Euthanasia, blood collection and necropsy

Bats were deeply anesthetized and maintained with 3% isoflurane and an oxygen flow rate of 1.5 L/min. Deep pain was assessed by firmly pinching skin and toes with forceps and assessed for any response. A thoracotomy was then performed with sterile standard scissors to puncture through the skin, muscle and diaphragm just caudal to the sternum and cut through the wall of the chest cavity caudally to cranially—removing and preventing negative pressure from building in the thorax.

Cardiac blood was collected with a 21 gauge sterile needle inserted into the apex of the heart. A maximum blood volume of between 1 and 1.5mls is collected in a syringe and transferred to a red top tube (RTT). RTTs sat at room temperature for one hour to allow a clot to form and then centrifuged at 1000 x g for 10 min at room temperature. Serum was removed from the clot, placed in a new microcentrifuge tube and stored at -20°C.

Serum from bats at 2 and 5 dpi were used to assess for viremia. Serum from 10 dpi and the 28 dpi pilot study bats were used to determine antibody titers. Because blood draws yield a small volume of blood (50 μl whole blood for a non-terminal blood draw, 500 μl whole blood for terminal blood draw) it was necessary to prioritize samples to optimize data retrieved. In order to assay the serum for viral RNA and perform serology, earlier time points were used to assess for viremia and later time points for seroconversion. Along with sample partitioning for data maximization, the small blood volume led to concerns that there would be an undetectably small viral load. To circumvent this issue, neat serum and 1:10 diluted serum were inoculated onto Vero cells to amplify any virus that may have been present at low levels. One blind passage on Vero cells was done and cell supernatants assayed by qRT-PCR. The remaining serum from three of the four bats was assayed directly for ZIKV RNA.

Necropsies were performed immediately after euthanasia. Bats were assessed for gross pathology. The following tissues were collected for both experiments: heart, lung, liver, spleen, kidney, urinary bladder, prostate, testes, and brain. A portion of tissues were collected and kept at -80°C for RNA extraction, and a portion placed in 10% buffered formalin for histology at a 1:10 weight to volume ratio for histology.

For a negative control animal a male bat was trapped from the colony and euthanized under the same protocol as the experimental infection bats.

### Serology

Vero E6 cells (ATCC) were propagated to 60% confluency in a 96-well tissue culture plate and infected with ZIKV strain PRVABC at an m.o.i. of 0.1. After a one hour incubation period, unbound virus was removed and replaced with 2% FBS-DMEM and incubated for a maximum of three days. Media was then replaced with 85% acetone for 20 minutes at -20°C to fix virus-infected cells to plate and serve as an antigen for enzyme-linked-immunosorbent assay (ELISA). Plates were stored at 4°C until use and used within two weeks. Plates were washed 5x with 0.05% Tween 20-PBS and blocked with SuperBlock T20 (TBS) Blocking Buffer (Thermo Fisher Scientific, Waltham, MA) for one hour at room temperature. Serum from an uninfected bat was used for a negative control. A convalescent human serum sample (kindly provided by B. Foy, CSU) was used as a positive control. A two-fold serial dilution was used starting at 1:100 to 1:12800. Diluted serum was placed in wells and incubated for two hours at room temperature. Serum was removed and plates washed. HRP-conjugated protein A/G (Thermo Fisher Scientific, Waltham, MA) was added at a concentration of 2 μg/ml to each well, and incubated for 30 minutes at room temperature. HRP-conjugated protein A/G was used in place of a secondary antibody as it targets the Fc portion of an antibody, which is highly conserved and therefore can be used for multiple animal species [54]. Plates were washed and 150 μl of ABTS Peroxidase Substrate (2 component) (KPL, Gaithersburg, MD) added according to manufacturers’ instructions, incubated at room temperature for 30 minutes, and then 150 μl of ABTS Peroxidase Stop solution (KPL, Gaithersburg, MD) added. Plates were read on an EMax Plus Microplate Reader (Cambridge Scientific, Watertown, MA). Absorbance was measured at 405 nm and the limit of detectable response was set at three standard deviation values above mean negative control serum.

### RNA extraction

TRIzol Reagent was used for RNA extraction from serum-cell supernatants, serum, urine and tissues according to Ambion, Life Technologies protocol. For tissues, approximately 50 mg of tissue was homogenized with one mL of TRIzol Reagent. A 5mm stainless steel bead (Qiagen, Valencia, CA) was used with a TissueLyser LT (Qiagen, Valencia, CA) at 50 Hz for 5 minutes. One ml of TRIzol was added to urine to 5 to 15 μl of urine. One ml of TRIzol was added to 160 μl of serum from AJ-z2, AJ-z3, and AJ-z4. Two-hundred microliters of serum-cell supernatants were added to one ml of TRIzol. Samples were then incubated at room temperature for 5 minutes. Chloroform (Thermo Fisher Scientific, Waltham, MA) was added, samples were mixed, incubated for 3 minutes at room temperature and centrifuged at 12,000 x g for 15 minutes at 4°C. The aqueous phase was removed, 4 μg of glycogen (Thermo Fisher Scientific, Waltham, MA) and 100% molecular grade isopropanol added (Thermo Fisher Scientific, Waltham, MA). Samples were incubated at room temperature for 10 minutes and then centrifuged at 12,000 x g for 10 minutes at 4°C. Supernatant was removed and 75% molecular grade ethanol (Thermo Fisher Scientific, Waltham, MA) was added to RNA pellet. Samples were vortexed and centrifuged at 7500 x g for 5 minutes at 4°C. Wash was removed and air-dried. RNA was resuspended in RNase-free water and stored at -80°C for future use.

### Viral RNA detection in serum samples

Vero cells were grown to 70 to 80% confluency in a 48-well tissue culture plate with 10% FBS-DMEM. Media was removed and 100 ul of bat serum from 2 dpi bats and 5 dpi bats was inoculated onto cells. Additionally, serum from each bat was diluted 10-fold in 2% FBS (Millipore Sigma) PBS supplemented with 1% calcium and magnesium, and inoculated onto cells. Samples were incubated for one hour at 37°C. Inoculum was removed and cells washed twice in sterile PBS. Two-percent FBS-DMEM was added to wells and plates were incubated at 37°C, 5% CO_2_. Cells were assessed daily for cytopathology (CPE) through day 7 but none was observed. Two-hundred microliters of the supernatant was removed on day 7 and used for RNA extractions. An additional 100 μl of supernatant was blind passaged onto Vero cells at 70 to 80% confluency. Cells were incubated for one hour at 37°C, washed twice with sterile PBS and 2% FBS-DMEM added. On day seven, supernatant was removed and TRIzol extractions performed for RNA recovery. Serum was treated as such in an attempt to amplify viral load and increase assay sensitivity serum may not be the most sensitive diagnostic sample [23–26].

If any serum was remaining it was directly used for TRIzol RNA extractions. Serum samples remained from AJ-z2 at 2 dpi, and AJ-z3 and AJ-z4 at 5 dpi. No serum remained from AJ-z1.

### Reverse transcription probe based real time PCR

Roche Real Time Ready RNA Virus Master Kit (Roche, Indianapolis, IN) was used on RNA extracted from serum-cell supernatants, serum, urine and tissue to assay for ZIKV RNA according to manufacturers’ instructions. Primers used were ZIKV 1086 (CCGCTGCCCAACACAAG) and ZIKV 1162c (CCACTAACGTTCTTTTGCAGACAT). Probe was ZIKV 1107-FAM (AGCCTACCTTGACAAGCAGTCAGACACTCAA) [55]. Two-hundred nanograms of sample RNA was added to each reaction. Reactions were performed in duplicate. Standards were a non-infectious clone of full length ZIKV strain PRVABC59 by which concentration was determined through optical density. Molecular weight of the genome sequence was used to calculate copy number [56]. A log_10_ dilution series of the standard was made and linear regression used to determine copy number equivalents of positive samples. Amplification was performed according to manufacturers’ protocol for Roche Real Time Ready RNA Virus Master Kit (Roche Diagnostics Corporation, Indianapolis, IN) with PCR conditions as follows: 8 min at 50°C, 30 s at 95°C, and 45 cycles of 10 s at 95°C, 20 s at 60°C and 10 s at 72°C.

### Histology

Tissues fixed in 10%-buffered formalin were cut in and submitted to Colorado State University Veterinary Diagnostic Laboratory (CSU VDL, Fort Collins, CO) for paraffin embedding, sectioning and staining with hematoxylin and eosin, as well as immunohistochemistry (IHC). Tissues cut in on bats to assess for histology included: heart, lung, liver, kidney, testes, prostate, urinary bladder and brain. Additionally, for AJ-z3 and AJ-z5 mandibular salivary gland was cut in. AJ-z4 had esophagus and lymphoid tissue that included palatine salivary gland cut in. Antibody for IHC was a polyclonal rabbit antibody that targets preM and E proteins of ZIKV and was provided by CSU VDL’s pathology department. The Bond-III automated instrument (Leica Biosystems, Wetzlar, Germany) was used for IHC staining. All slides were blindly read by a diplomat of the American College of Veterinary Pathologists.

### Tissue preparation for immunohistochemistry and immunofluorescence

Brain tissues was prepared for immunohistochemical and immunofluorescence staining as previously reported [57]. Tissue was dehydrated by using a graded ethanol series of 70% ethanol for 2 h, 80% overnight, 90% for 2 h and 100% for 2 h. Brain tissues were then post-fixed in dimethylbenzene for 30 min and embedded in dimethylbenzene-paraffin at 60°C for 2 h, after which samples were embedded in a metal frame. Sagittal sections were collected at 5um thick. All dewaxing, antigen retrieval and immunofluorescence staining was automated using a Leica Bond RXM. In short, sections were dewaxed using ethanol and then boiled in antigen retrieval solution for 10 minutes. The cooled sections were incubated in 3% H2O2 for 15 min at room temperature and then blocked with 2% donkey and goat serum (Millipore Sigma) for 1 hour. Rabbit anti-Iba1 (Wako Chemicals USA, Irvine, CA) and 4G-2 Flavivirus E specific monoclonal antibodies (CDC, Fort Collins) were diluted in TBS to final concentrations of 1:250 and 1:50, respectively. Sections were incubated in primary antibodies concurrently at room temperature for one hour. Following removal of unbound primary antibodies by washing, goat anti-rabbit secondary (AlexaFluor-555) and donkey anti-mouse secondary (AlexaFluor-647) was added and incubated for 1 hour at room temperature. Finally, DAPI counterstain (Vector Laboratories, Burlingame, CA) was applied and sections were washed with TBS prior to cover slipping for imaging.

### Confocal microscopy

Stained sections were imaged on a Ziess LSM 800 with Airyscan laser-scanning confocal microscope (Ziess, Oberkochen, Germany) using a 63× oil immersion objective. Each field of view was imaged as a *z*-stack (8–10 planes, .5-*μ*m step size) transformed into a single maximum projection image using the Ziess Zen (blue) imaging software.
